# Humanistic nursing care and quality of life in patients with nasopharyngeal carcinoma: a prospective observational study

**DOI:** 10.1186/s12912-026-04455-9

**Published:** 2026-02-26

**Authors:** Cong Wang, Yanhua Xiao, Liping Wu, Yan He, Min Chen, Wen Hu, Yuying Fan

**Affiliations:** 1 Department of Nasopharyngeal Carcinoma, Sun Yat-sen University Cancer Center, Guangzhou, China; 2 State Key Laboratory of Oncology in South China, Guangdong Provincial Clinical Research Center for Cancer, Collaborative Innovation Center for Cancer Medicine, Guangdong Key Laboratory of Diagnosis and Therapy, 651 Dongfeng Road East, Guangzhou, 510060 China

**Keywords:** Nasopharyngeal carcinoma, Nursing humanistic care, The quality of life

## Abstract

**Purposes:**

To study the relationship between nursing humanistic care and quality of life in patients with nasopharyngeal carcinoma (NPC).

**Methods:**

A total of 382 NPC patients of Sun Yat-sen University Cancer Center were included from October 1, 2018, to July 30, 2019. The Nursing Humanistic Care Experience Scale-Noninfectious Chronic Disease Patients version (NHCES-NCDP) and quality of life questionnaire of the European Organisation for the Research and Treatment of Cancer (EORTC) QLQ-C30 (V3.0)、QLQ—H&N35 (V1.0) were used to assess the relationship between the score of nursing humanistic care and quality of life. Paired sample T-test, Pearson correlation test and linear regression were used to analyse the data on the relationship between nursing humanistic care and the quality of NPC patients.

**Results:**

The score of humanistic care post-treatment in NPC patients was higher than that of pre-treatment (101.4 ± 16.6 vs. 99.3 ± 16.8); The scales of cognitive functioning, social functioning, financial difficulties, nausea and vomiting among quality of life were significantly associated with the scores of Nursing Humanistic Care before treatment. Emotional functioning, Cognitive functioning, Social functioning, Fatigue, Nausea and vomiting, Insomnia, Appetite loss, Financial difficulties, and pain among quality of life were significantly associated with Nursing Humanistic Care after treatment. Still, there was no correlation among other items.

**Conclusion:**

The quality of life score was reduced after chemo-radiotherapy in NPC patients, and it was significantly associated with humanistic nursing care. The quality of life of NPC patients may be improved by elevating the nursing humanistic care level.

**Clinical trial number:**

Not applicable.

**Supplementary Information:**

The online version contains supplementary material available at 10.1186/s12912-026-04455-9.

## Background

Nasopharyngeal carcinoma (NPC) has typical regional characteristics and is one of the most common cancers in Southeast China and East Asia [1]. With the progress of treatment and the improvement of medical level, the cure rate of nasopharyngeal carcinoma is improving year by year, and the mortality rate is going down sharply [1, 2]. However, there are many side effects in NPC patients during the treatment, especially concurrent chemoradiotherapy [3], such as oral mucositis, nausea, vomiting, pain, weight loss, neck fibrosis, dysphagia, and xerostomia [4, 5]. Previous research showed that malnutrition and weight loss affect the prognosis of nasopharyngeal cancer in turn [6, 7]. These conditions coupled with under-appreciated symptom management and fragmented external support systems, magnify difficulties in the medical experience. Therefore, it is essential to enhance the humanistic nursing care for NPC patients and pay attention to the changes in their physiological and psychological conditions.

It is necessary to pay attention to the quality of life in NPC patients. With the development of current health concepts and medical models, more and more attention is paid to the quality of life at different levels of physiology, psychology and society. And it has become an important outcome index to evaluate the effect of cancer treatment [8]. By assessing the quality of life of patients, clinicians can not only understand the health status of different patients, but also choose different treatment methods and drugs as a therapeutic effect evaluation index, which has good social benefits [9]. Besides, the use of appropriate scales is the key to measuring patients’ quality of life. Currently reported Cancer scale systems include the Functional Assessment of Cancer Therapy (FACT) in the United States and the European Organisation for Research and Treatment of Cancer (EORTC) QLQ scale system.

Humanistic care is putting the concept of “patient-centred” service into daily clinical practice to truly serve patients [10]. With the development of “High-Quality Nursing Service,” the Patient-centred service concept is becoming increasingly important in nurses’ clinical work [11]. It facilitates patients’ ability to make health-related decisions and promotes an environment of developing a model of clinical practice that is compassionate, caring, respectful, and kind to patients [11, 12]. Rather than acting as a direct determinant of quality of life, humanistic care is theorised to affect quality of life indirectly through psychosocial pathways, particularly emotional support, trust in care providers, and perceived involvement in care.

Many factors influence humanistic care. On the one hand, the ability of humanistic care is affected by gender, the nature of the department/division, and modes of employment [12]. The humanistic ability of female or male doctors/nurses is different. Environment, holistic care, individualisation, and autonomy have also influenced the experience of nursing humanistic care in hospital patients [13]. Nurses in China struggle with the large clinical workload with fewer resources, resulting in decreased job satisfaction, the lack of humanistic care for patients, and patients’ interpersonal relationships, social service, and nursing environment [14, 15]. The humanistic care of nursing is still in the developing stage, and its quality control needs to be improved.

These observations highlight the multifaceted care needs of NPC patients beyond tumour control alone. Given this multifaceted symptom burden and psychosocial complexity, conventional biomedical care alone may be insufficient to address the full aspect of patient needs [16, 17]. Humanistic nursing care emphasises empathy, communication, emotional support, and engagement with patients, which may therefore be particularly important in NPC care. It tries to address both physical and psychosocial dimensions of patient experience. This suggests that humanistic nursing care could play a vital role in addressing not only physical symptom burden but also emotional well-being and resilience.

Previous study [18] reported that humanistic care interventions for cancer patients had a positive effect on a good state of mind, better control of pain, the quality of sleep and a positive nurse-patient relationship, while the participants of this study did not include NPC patients. The relationship between nursing humanistic care and the quality of life of NPC patients had not been studied before. Therefore, this study will focus on the quality of life of NPC patients during treatment and humanistic nursing care for NPC patients. This study aims to explore the correlation between nursing humanistic care and the quality of life in NPC patients.

Therefore, this study aimed to address the following research questions:

1. What is the level of perceived nursing humanistic care among NPC patients before and after treatment?
2. How is humanistic nursing care associated with quality-of-life outcomes in NPC patients?
3. Which domains of quality of life are most closely related to humanistic care during treatment in NPC patients?

## Methods

### Participants

A convenience sample of 382 Chinese nasopharyngeal carcinoma patients of Sun Yat-sen University Cancer Centre was selected from October 2018 to July 2019. Inclusion criteria: (1) Participants were required to have nasopharyngeal carcinoma as diagnosed by pathology for the first time, (2) age between 18 and 70 years, (3) the NPC patient is ready to start receiving anti-tumthis treatment. administered either on an outpatient basis or during hospitalization. (4) agreement to participate in this study, independently or with family members’ help. Two of the participants were illiterate, and their family members helped them to fill in the questionnaire by reading the questions to them in turn. Exclusion criteria: (1) severe physical diseases, such as severe cardiopulmonary disease, liver and kidney failure, and severe immune deficiency that cannot tolerate radiotherapy, chemotherapy or major surgery, (2) terminal stages of the disease, (3) mental illnesses and cannot fill in questionnaires morally. (4) have previously received systemic chemoradiotherapy or other experimental treatments. Eligible patients were consecutively recruited during the study period.

### Sample size

The sample size was estimated according to the estimation principle proposed by Kendall. The sample size was 5 to 10 times the number of variables. In this study, the number of independent variables involved was 24, and the baseline characteristics were 8. Therefore, at least 160 cases need to be included. Considering a 20% dropout rate and a 5% mortality rate, the sample size should be at least 200. This study intends to include 382 patients.

### Questionnaire

The nursing humanistic care was assessed by using the Nursing Humanistic Care Experience Scale for Noninfectious Chronic Disease Patients (NHCES-NCDP), a tool to measure nurse-patient relationships from a patient’s perspective [19]. The scale was constructed by carrying out the translation of the Human Relations Experience Scale (HRES) developed by Boscar for patients with chronic diseases [20]. Good reliability and validity have been reported for the Chinese version of NHCES-NCDP, the Cronbach α coefficient was 0.965, and the internal measure reliability was 0.710 [21]. NHDCES-NCDP includes 24 items, and each item is scored using a five-level Likert scale,1 = never, 2 = rarely, 3 = sometimes, 4 = often, and 5 = always. The 24 questions are grouped into three composite scales: Positive relationship building, Problems 1 through 4, 9 through 11; Approval and support of choice, Problems 12,14 through 24; Promoting comfort, Problems 5 through 8,13 [21]. The score for every composite item is the sum of the scores for each item it contains. The higher each score, the better nursing humanistic care that patients experience, and the higher the quality of nursing humanistic care [22].

The European Organisation for Research and Treatment of Cancer (EORTC) Study Group has developed the quality of life questionnaire core-30 (EORTC QLQ-C30) [23]. The QLQ-C30 is a quality-of-life instrument for cancer patients, which has been used in a wide range of cancer clinical trials by a large number of research groups. The simplified Chinese version of EORTC QLQ-C30 has been applied in China and proved to have high reliability (0.7) and validity (0.5) [24]. The QLQ-C30 comprises 30 items including five functional scales(physical, role, emotional, cognitive, and social functioning), three symptom scales(fatigue, nausea and vomiting, and pain), a global health status scale, and six single items (dyspnea, insomnia, appetite loss, constipation, diarrhoea, and financial difficulties) [25]. All of the scales and single-item measures range in scores from 0 to 100. A higher score for a functional scale and the global health status represents better functioning and quality of life for patients. A higher score for a symptom scale represents worse QoL.

As reported in previous studies, the EORTC quality of life questionnaire in head and neck cancer (QLQ–H&N35) comprises 35 questions assessing symptoms and side effects of treatment, including seven multi-item scales (pain, swallowing, senses (taste and smell), speech, social eating, social contact, and sexuality) and eleven single items (teeth, opening mouth, dry mouth, sticky saliva, coughing, felt ill, pain killers, nutritional supplements, feeding tube, weight loss, weight gain) [26]. The QLQ-H&N35 was used in the evaluation of quality of life in NPC patients [27]. The scoring approach for the QLQ-H&N35 is identical in principle to that for the symptom scales of the QLQ-C30. For all items and scales, high scores indicate more problems and low QoL [29].

### Data collection

Data collection followed a standardised procedure applied uniformly to all participants. This study is a cohort study, and a convenience sampling strategy was used. Data were collected in both the outpatient treatment area and the inpatient wards of the Department of Nasopharyngeal Carcinoma at Sun Yat-sen University Cancer Center. The trained research nurse issued the questionnaires of the Humanistic Care Experience Scale (NHCES-NCDP), EORTC QLQ-C30, and the QLQ-H&N35 before and after treatment, and checked the questionnaires to make sure they were completed. Recorded using a standardised data collection form (see Additional file 1). Every patient had filled in these three questionnaires within 15–20 min at two time points, pre-treatment and post-treatment. The first examination refers to the time, which is around one week, before chemoradiotherapy (pre-treatment). The second examination refers to the time, which is around one week, after participants received chemoradiotherapy (post-treatment). 395 questionnaires were handed out, and 100% were returned. Among them, 13 questionnaires were invalid due to many missing questions. So the response rate was 97% (382/395). Informed consent was obtained from the patients before the research.

### Ethical considerations

This study was approved by the Ethics Committee of Sun Yat-sen University Cancer Center. Written informed consent was obtained from all participants before enrollment. Participants were informed about the purpose of the study, the voluntary nature of participation, and their right to withdraw at any time without consequences for their medical care. All data were anonymised, stored securely, and used solely for research purposes to ensure confidentiality.

### Statistical analysis

The Statistical Package for the Social Sciences (SPSS) version 23.0 was used for the statistical analyses. Enumeration data are expressed in frequency and percentage. The numerical data were expressed as (mean ± SD). The score of quality of life before and after treatment was assessed by the paired sample T-test. The chi-square test was used for comparison between groups. Pearson correlation test and linear regression were used for the relationship between nursing humanistic care and quality of life. *p* < 0.05 and *p* < 0.001 were considered to be statistically significant.

## Result

The baseline characteristics of NPC patients and their QOL were collected and analysed in terms of age, sex, marital status, education level, work status, permanent residence, and treatment modalities. We found that 91.9% (351/382) patients were less than 60 years old 8.1% (31/382) were more than 60 years old, 72.5% (277/382) were male and 27.5% (105/382) were female, the QOL between different age and sex showed no significant differences (*p* = 0.614 and *p* = 0.332, respectively). There were no significant differences regarding marital status (*p* = 0.617), education level (*p* = 0.625), work status (*p* = 0.56), and permanent residence (*p* = 0.873). There were three types of medical insurance (e.g., commercial insurance, medical insurance, and social basic insurance), and the QOL scores of different insurances showed no significant differences (*p* = 0.426). Regarding treatment modalities, 170 (44.5%) patients received induction plus concurrent chemotherapy, and 212 (55.5%) received Concurrent chemotherapy radiotherapy, and the QOL score of patients in the two treatments showed no significant differences (*p* = 0.75). As shown in Table 1.

**Table 1:** Baseline characteristics and univariate analysis for QoL of NPC patients (*n* = 382)

characteristics	*N*(%)	QOL(M ± SD)	F/t	*P*
Sex			0.992^1)^	0.332
male	277(72.5)	69.28 ± 21.74		
female	105(27.5)	66.83 ± 22.31		
Age			0.488^2)^	0.614
18–45	222(58.1)	69.51 ± 21.91		
46–60	129(33.8)	67.50 ± 22.24		
≥ 61	31(8.1)	66.67 ± 16.67		
Marital status			0.50^1)^	0.617
married	362(94.8)	68.74 ± 21.61		
others	20(5.2)	66.25 ± 22.37		
Education level			0.653^2)^	0.625
illiteracy	2(0.5)	50.00 ± 23.57		
Primary	38(9.9)	68.64 ± 20.73		
Middle school	172(45.0)	67.49 ± 21.75		
High and technical secondary school	39(10.2)	69.44 ± 23.44		
University/college or above	131(34.3)	70.10 ± 21.25		
Employment status			0.58^2)^	0.56
employment	323(84.6)	69.18 ± 21.98		
unemployed	34(8.9)	65.93 ± 19.29		
retired	25(6.5)	65.67 ± 20.17		
Place of residence			0.905^1)^	0.873
rural	193(50.5)	67.62 ± 21.60		
urban	189(49.5)	69.62 ± 21.67		
Medical fee			0.931^2)^	0.426
Commercial Insurance	6(1.6)	68.06 ± 17.01		
Medical insurance	362(94.8)	68.26 ± 21.77		
Social Basic insurance Self-pay	13(3.4) 1(0.3)	77.56 ± 19.06		
Treatment			-1.335^1)^	0.75
Induction chemotherapy and concurrent chemotherapy	170(44.5)	66.96 ± 21.79		
Concurrent chemoradiotherapy	212(55.5)	69.93 ± 21.45		

1) = t; 2) = F NPC: nasopharyngeal carcinoma

Compared with pre-treatment, the score of the humanistic care experience scale after treatment had significant differences in NPC patients (*P* = 0.013). The scores were significantly different between “the positive relationship building” and “approval and support of choice” before and after treatment in NPC patients (*P* < 0.001, *P* = 0.002). The scores of “promoting comfort” were higher than the scores of the other two items of “positive relationship building” and “approval and support of choice”. The scores of “promoting comfort” showed no statistically significant differences before and after treatment in NPC patients(*P* = 0.787), as shown in Table 2.

**Table 2:** The score of NHCES before and after treatment in NPC patients(*n* = 382)

Items(Mean ± SD)	Pre-treatment	Post-treatment	*P*
The positive relationship building	27.70 ± 5.76	29.02 ± 5.54	< 0.001
Approval and support of choice	20.27 ± 4.12	20.93 ± 3.90	0.002
Promoting comfort	51.35 ± 8.16	51.47 ± 8.38	0.787
Global score	99.33 ± 16.81	101.42 ± 16.62	0.013

NHCES: Nursing Humanistic Care Experience scale

NPC: nasopharyngeal carcinoma

The score of global health after treatment in NPC patients was lower than the score of global health before treatment (57.42 ± 21.23 vs. 68.61 ± 21.60, *p* < 0.001) in the QLQ-C30. The score of physical functioning was the highest (88.48 ± 13.50), while the score of social functioning was the lowest (71.55 ± 19.36) before treatment in functional scales. The score of financial difficulties was the highest (35.25 ± 33.06), while the score of diarrhoea was the lowest (6.54 ± 14.52) before treatment in symptom scales. The score of cognitive functioning was the highest (75.87 ± 20.89), while the score of social functioning was the lowest (67.06 ± 24.88) after treatment in the functional scales of NPC patients. The score of cognitive functioning was the highest (75.87 ± 20.89), while the score of social functioning was the lowest (67.06 ± 24.88) after treatment in functional scales. The score of Appetite loss was the highest (50.09 ± 27.82), while the score of diarrhoea was the lowest (6.98 ± 15.01) after treatment in the symptom scale. All the scores of function scales after treatment were significantly lower than the scores before treatment (*P* < 0.001), and all the scores of symptom scales after treatment were significantly higher than the scores before treatment (*P* < 0.001), except diarrhoea and financial difficulties. As shown in Table 3.

**Table 3:** The score of QLQ-C30 before and after treatment in NPC patients(*n* = 382)

Subscales(Mean ± SD)	Pre-treatment	Post-treatment	*P*(T-test)
Global health status	68.61 ± 21.60	57.42 ± 21.23	< 0.001
Physical functioning	88.48 ± 13.50	71.55 ± 19.36	< 0.001
Role functioning	86.91 ± 20.57	68.46 ± 25.85	< 0.001
Emotional functioning	80.96 ± 17.90	73.89 ± 20.21	< 0.001
Cognitive functioning	87.22 ± 15.42	75.87 ± 20.89	< 0.001
Social functioning	73.21 ± 23.96	67.06 ± 24.88	< 0.001
Fatigue	22.43 ± 19.51	44.21 ± 22.75	< 0.001
Nausea and vomiting	11.95 ± 19.46	36.43 ± 25.41	< 0.001
Pain	15.01 ± 19.17	38.52 ± 26.70	< 0.001
Dyspnoea	10.21 ± 17.68	20.51 ± 22.16	< 0.001
Insomnia	21.17 ± 24.43	36.73 ± 27.59	< 0.001
Appetite loss	18.15 ± 24.79	50.09 ± 27.82	< 0.001
Constipation	12.49 ± 20.45	30.19 ± 26.04	< 0.001
Diarrhoea	6.54 ± 14.52	6.98 ± 15.01	0.660
Financial difficulties	35.25 ± 33.06	37.52 ± 32.13	0.170

QLQ-C30: the EORTC quality of life questionnaire C30

NPC: nasopharyngeal carcinoma

The scores of multi-items and single items of QLQ-H&N35 are shown in Table 4. The symptoms and problems after treatment were significantly different from those before chemoradiotherapy in NPC patients (*P* < 0.001). The 7 symptoms scares of pain, swallowing, sense problems, speech problems, trouble with social eating, trouble with social contact, and less sexuality, and 9 single symptoms of teeth, opening mouth, drying mouth, sticky saliva, coughing, feeling ill, pain killers, nutritional supplements, feeding tube, weight loss, were increased significantly after treatment than that before treatment in NPC patient (*P* < 0.001). The score of weight gain after treatment (4.71 ± 21.22) was significantly higher than the score (14.14 ± 34.89) before treatment (*p* < 0.001). The score of weight loss (34.82 ± 48.25) was highest, while the score of feeling tube (4.19 ± 20.06) was lowest before treatment in NPC patients. The score of weight loss (92.67 ± 26.10) was highest after chemoradiotherapy in NPC patients.

**Table 4:** The score of QLQ-H&N35 before and after treatment in NPC patients(*n* = 382)

Subscales(Mean ± SD)	Pre-treatment	Post-treatment	*P*(T-test)
Pain	8.59 ± 12.60	44.04 ± 25.33	< 0.001
Swallowing	4.65 ± 11.38	42.26 ± 26.62	< 0.001
Senses problems	9.25 ± 14.75	47.12 ± 26.05	< 0.001
Speech problems	6.05 ± 11.84	26.32 ± 22.11	< 0.001
Trouble with social eating	6.70 ± 12.72	34.75 ± 21.10	< 0.001
Trouble with social contact	6.30 ± 11.44	24.66 ± 21.51	< 0.001
Less sexuality	21.03 ± 24.80	40.75 ± 31.54	< 0.001
Teeth	13.79 ± 20.40	22.51 ± 25.66	< 0.001
Opening mouth	7.07 ± 15.63	31.59 ± 27.80	< 0.001
Dry mouth	21.90 ± 22.79	53.32 ± 28.15	< 0.001
Sticky saliva	16.23 ± 20.87	53.66 ± 29.92	< 0.001
Coughing	12.69 ± 19.48	32.90 ± 28.35	< 0.001
Felt ill	25.92 ± 25.10	41.97 ± 28.85	< 0.001
Pain killers	9.86 ± 26.21	43.46 ± 69.84	< 0.001
Nutritional supplements	29.58 ± 45.70	62.83 ± 48.39	< 0.001
Feeding tube	4.19 ± 20.06	8.38 ± 27.74	0.013
Weight loss	34.82 ± 48.25	92.67 ± 26.10	< 0.001
Weight gain	14.14 ± 34.89	4.71 ± 21.22	< 0.001

NPC: nasopharyngeal carcinoma

QLQ-H&N35: the EORTC quality of life questionnaire of head and neck

The correlation between nursing humanistic care and QLQ-C30 of NPC patients before treatment is shown in Table 5. The cognitive functioning (R^2^ = 0.156, *P* = 0.002), social functioning (R^2^ = 0.129, *P* = 0.011), nausea and vomiting (R^2^=-0.109, *P* = 0.034) and financial difficulties (R^2^=-0.143, *P* = 0.005) were significantly correlated with nursing humanistic care before treatment in NPC patients. However, there was no significant correlation between nursing humanistic care and other scales of QLQ-C30 and QLQ-H&N35 before treatment in NPC patients.

**Table 5:** Correlation of the NHCES with EORTC QLQ-C30 and QLQ-H&N35(*n* = 382)

QLQ-C30 subscales	NHCES		QLQ-H&N35 subscales	NHCES	
	*R* ^2^	*P*		*R* ^2^	*P*
cognitive functioning(pre)	0.156	0.002	Pain(post)	-0.124	0.015
social functioning(pre)	0.129	0.011	Trouble with social eating(post)	-0.177	< 0.001
nausea and vomiting(pre)	-0.109	0.034	Trouble with social contact(post)	-0.177	< 0.001
financial difficulties(pre)	-0.143	0.005			
emotional functioning(post)	0.182	< 0.001			
cognitive functioning(post)	0.138	0.007			
social functioning(post)	0.127	0.013			
fatigue(post)	-0.156	0.002			
nausea and vomiting(post)	-0.13	0.011			
Insomnia(post)	-0.139	0.006			
Appetite loss(post)	-0.116	0.023			
financial difficulties(post)	-0.171	0.001			

Pre: pre-treatment/before treatment; Post: post-treatment/after treatment;

R^2^: Pearson correlation test

QLQ-H&N35: the EORTC quality of life questionnaire of head and neck 35

QLQ-C30: the EORTC quality of life questionnaire C30

NHCES: Nursing Humanistic Care Experience scale

It is shown that nursing humanistic care was significantly correlated with emotional functioning, cognitive functioning, social functioning, fatigue, nausea and vomiting, Insomnia, appetite loss, and financial difficulties of QLQ-C30 after chemoradiotherapy in NPC patients (*P* < 0.05) in Table 5. Meanwhile, there was also a relationship related to nursing humanistic care, such as pain, trouble with social eating, and trouble with social contact of QLQ-H&N35 after treatment. There were no significant differences between NHCES and other scales/items of QLQ-H&N35.

Overall, the score of Global health status was the dependent variable for patients, general and clinical characteristics, the score of Global health status before treatment, 、the score of NHCES before and after treatment are the independent variables, and linear regression analysis is conducted. The results showed that quality of life and quantity have no relationship with humanistic care (*P* > 0.05), as shown in Table 6.

**Table 6:** Linear regression of Global health status (post-treatment) with general and clinical characteristics and NHCES score(*n* = 382)

Factors	Regression coefficient	Standard error	Regression coefficient	t	*P*
NHCES (pre)	-0.233	0.079	-0.181	-2.947	0.003
NHCES (post)	0.239	0.079	0.187	3.033	0.003
Sex	-4.177	2.565	-0.087	-1.628	0.104
Age	-4.248	2.328	-0.127	-1.825	0.069
Education level	-1.944	1.171	-0.097	-1.660	0.098
work	5.191	2.540	0.137	2.044	0.042
married	4.838	5.308	0.048	0.911	0.363
Medical insurance	-1.231	4.142	-0.016	-0.297	0.767
Permanent residence	0.063	2.419	0.001	0.026	0.979
Treatment	5.817	2.287	0.135	2.544	0.011

Pre: pre-treatment; Post: post-treatment/after treatment;

NHCES: Nursing Humanistic Care Experience scale

## Discussion

This study addressed previously unexplored questions regarding the relationship between humanistic nursing care and quality of life in NPC patients. The findings help clarify how nursing humanistic care relates to the psychosocial and functional aspects of quality of life during treatment. There are a few articles on nurse humanistic care at present that construct a knowledge, attitude, and practice evaluation system of nursing humanistic care in China. Previous studies suggest that building trust with diabetic patients is important to achieve favourable treatment outcomes [28]. However, there have been no articles on humanistic care for NPC patients. Researching concepts related to “nursing humanistic care”, such as humanistic care, humanistic nursing, humanistic model of care, and humanistic care practice in the PubMed databases. This is helpful to search for more evidence in the foreign language database. Lu et al. [29] and Gao et al. [29] investigated the influence of humanistic care on nursing quality and patient psychology of advanced non-small cell lung cancer. However, the outcome is pain, anxiety and depression, and there were no physical functions. Besides, the participants are lung cancer patients and ovarian cancer patients, but not NPC patients. The findings suggest that humanistic nursing care may be particularly relevant in NPC, who suffer high symptom burden, and sustained psychosocial needs. The study is different from the two mentioned above and observes all aspects of quality of life in NPC patients. All in all, it is necessary to explore the relationship between the quality of life of NPC patients and nurse humanistic care.

Nursing quality in cancer care is multidimensional and encompasses technical competence, symptom management, self-care support, and relational care. In this study, humanistic care represents one relational dimension of nursing quality rather than a comprehensive measure of overall nursing performance. The researchers examined the relationship between nurses’ humanistic care and the quality of life of NPC patients before and after treatment. Although the data were collected between 2018 and 2019, the humanistic nursing practices evaluated in this study represent routine care processes that have remained largely unchanged, supporting the continued relevance of our findings.

Higher nursing humanistic care scores are associated with better quality of life and patient experiences in NPC patients. Previous research highlighted that the demands for humanistic care are positively correlated with psychological well-being and the ability to cope with illness among cancer inpatients, underscoring the integral role of humanistic nursing in addressing psychological distress and overall patient needs [30]. A study patient-patient-centred communication demonstrated that enhancing nurse–patient interactions improves patients’ perceptions of care quality, which are fundamental elements of high-quality nursing practice [31]. Person-centered care frameworks and patient experiences in oncology also highlight the value of tailoring care to individual preferences and needs to improve overall care experiences and outcomes [32]. These studies are consistent with findings and indicate that humanistic care may contribute to improving patient experience and quality of life.

This study showed a positive association between nursing humanistic care and QLQ-C30 subscales of different functioning, which includes cognitive functioning, social functioning, and emotional functioning. Nursing humanistic care was negatively associated with symptoms of NPC patients, including nausea and vomiting, fatigue, insomnia, appetite loss, pain, and trouble with social eating and contact. This suggests that a positive humanistic care experience is associated with better quality-of-life outcomes in NPC patients during chemoradiotherapy. This study insists that it is valuable to strengthen nurse humanistic care for NPC patients. Strengthening humanistic nursing care may be of clinical relevance in improving patient experience and psychosocial aspects of quality of life in NPC patients. The findings are consistent with previous studies demonstrating that humanistic and person-centred nursing practices improve patients’ emotional well-being, enhance nurse–patient relationships, and contribute to better overall care experiences [33–36].

Nurses provide high-quality humanistic care to NPC patients relatively. The score of NHCES-NCDP is higher after treatment than the score before treatment, and the total score is 120. These findings indicate that patients reported a relatively high level of perceived nursing humanistic care, which is consistent with the study of Hu et al. [37] This can be explained in the following ways: Nurses will introduce the doctor and nurse in charge, the environment of words, some suggestions and precautions for treatment in detail, and so on to NPC patients. Patients need to reduce the strangeness and adapt to the treatment process before treatment. Some patients are full of worry and fear about chemoradiotherapy. Nurses explain the illness and give psychological support at the same time, which helps to reduce the psychological pressure on them. So, the score of NHCES-NCDP after treatment is higher than before treatment. However, there were no statistically significant differences between the scores of NHCES-NCDP before and after treatment. That suggests that the nursing humanistic care needs to be further strengthened. Firstly, chemotherapy could cause a series of side effects for NPC patients, such as nausea and vomiting, pain, and fatigue. As they suffer great physical discomfort, they may need more humanistic care. Secondly, Nurses are usually busy with their clinical work, which occupies a lot of time, so they may neglect their care for patients. The nurse should pay more attention to the psychological changes of patients who receive radiation and chemotherapy. It is helpful for patients if nurses give more understanding and encouragement to patients who experience adverse reactions and symptoms during the treatment.

The results showed that the scores were significantly different between “the positive relationship building” and “approval and support of choice” before and after treatment, respectively. These suggest that nurses and patients can establish more harmonious relationships after treatment. During hospitalisation, Nurses play an important role between doctors and patients, and the nurse and the patient become more familiar with each other as the treatment progresses. Nurses should not only care about patients during treatment but also respect patients’ privacy and choice. The trust between nurses and patients maintains a harmonious personal relationship. However, there were no statistically significant differences in the promotion of comfort of NPC patients before and after treatment, and this may be due to the patient’s physical discomfort and chemotherapy adverse reactions.

The quality of life of NPC patients who have undergone radiotherapy and chemotherapy has reduced sharply. The scores of all the function scales were.

Radiotherapy hurts the patient’s health, emotions, and daily social interaction. Cisplatin is a chemotherapy drug for NPC patients, which causes gastrointestinal reactions such as decreased appetite, nausea and vomiting, abdominal distension, oral mucositis, and so on. Otherwise, a sore throat and radiation dermatitis also occurred due to radiotherapy. The symptoms became more severe than before with the number of radiotherapy sessions. The patient’s throat became so sore that it was difficult to eat after 15 sessions of radiation. Furthermore, patients may experience a variety of symptoms of radiation-induced dermatitis, such as pruritus, dryness, erythema, desquamation, necrosis, and ulceration, which affect patients’ comfort or even discontinuation of radiotherapy [33]. The doctor and nurse should not only care about the treatment effect but also pay attention to the quality of life of NPC patients. The score of appetite loss is the highest on the symptom scale, as the main side effect of chemotherapy is adverse reactions in the gastrointestinal tract. About two-thirds of cancer patients experience taste changes, and the common taste disorders are the “bitter” and “sweet” qualities of taste [38]. Bloating, acid reflux, nausea, and vomiting affect appetite adversely, reducing the patient’s food intake. This study reported that the quality of life of NPC patients improved immediately after treatment.

The social functioning of NPC patients is not optimistic. The score of social functioning is lower than the other scales of functioning before and after treatment. This suggests that the patient’s burden of social and psychological issues is apparent, and the functioning of society is affected by NPC. Feel depression, anxiety, and other emotional problems after the NPC is confirmed. They had to give up some family and social roles to receive treatment, and the normal course of life was disturbed. Family connections may be the only social relationships throughout diagnosis and treatment in some cases, so the social function level is lower in NPC patients. On the other hand, the patient’s social dimension and the main aspects of support are important and can determine how to face cancer [39]. A previous study indicated that a high level of positive emotions and the ability of patients are helpful to all family members, which could reduce the anxiety and stress of the family [40]. Emotional support from friends and family also plays a vital role in patients’ return to society [41]. Increasing the sense of belonging to a family and commonality with the family helps reduce stress. Assessing and enhancing family resilience might be crucial for the improvement of social functioning [40]. The physical function is good (88.5 ± 13.5) in NPC patients before treatment (the total score is 100), whose score of KPS is over 90. This may be related to the prime age of NPC patients. 30–45 years old is the most common age for NPC patients, and it has a younger trend. Those who have better physical quality than the old can take care of themselves in their daily life, and it is not difficult to do some physical work and sports.

Higher levels of perceived humanistic care were associated with better emotional functioning and lower levels of insomnia, fatigue, and appetite loss during the post-treatment period. The quality of life in NPC patients is multifactorial and shaped by disease severity, treatment-related toxicities, functional impairment, and social support. In this context, humanistic nursing care should not be viewed as an isolated determinant, but rather as a complementary and modifiable care component that may alleviate psychosocial distress and enhance patients’ coping and care experience during treatment. The results of this study indicate that, during the pre-treatment period, there is no significant correlation between patients’ emotional functioning, insomnia, fatigue, and appetite loss and humanistic care. However, there is a significant correlation between patients’ emotional functioning, insomnia, fatigue, and appetite loss and humanistic care during the post-treatment period. Moreover, compared to its effects on other physical functions and symptoms, humanistic care showed a stronger association with emotional functioning, insomnia, fatigue, and loss of appetite. This can be explained as follows: Before anti-cancer treatment, patients did not exhibit physiological symptoms such as fatigue or appetite loss. And the timing, intensity, or intervention model of humanistic care was insufficient to produce effects during the early stages of treatment. However, after patients underwent chemoradiotherapy or other treatments, they experienced emotional decline, physical fatigue, exhaustion, and appetite loss. Patients were in a state of high anxiety, which made emotional support and humanistic care particularly important. This is similar to the results of Cheung, who suggested that oncology care teams with humanistic care can mitigate disease impacts on mental health and social support, as well as employment or financial support services [42]. A previous study shows that humanistic care significantly reduces negative emotions and enhances comfort in women undergoing outpatient gynaecological surgery [43]. Besides, Savard claims that psychological cognitive intervention has shown significant improvements for cancer patients suffering from insomnia [44]. However, this study only discusses correlations between humanistic care and emotional function, insomnia, fatigue, and reduced appetite, and cannot directly infer causal relationships. We will also study further whether humanistic care improves symptoms and quality of function mentioned above, rather than merely being associated.

This study has a few limitations. Firstly, this study only selected two time points to study nurse humanistic care and quality of life of NPC patients, which are pre-treatment and post-treatment. However, the middle time point during cancer treatment is neglected. Secondly, it will be better to consider concepts related to “nursing humanistic care”, such as humanistic care, humanistic nursing, humanistic model of care, and humanistic care practice. It is helpful to search for more evidence-based information in the foreign language database. Thirdly, Potential sources of bias include convenience sampling, self-reported data, and single-centre recruitment, which may introduce selection and reporting bias. As pre-treatment and post-treatment humanistic care scores were derived from the same participants, potential collinearity between repeated measures cannot be excluded. Symptoms related to chemoradiotherapy often peak during treatment stages. Future studies, including multiple treatment time points, are needed to discuss symptom trajectories and care needs.

## Conclusion

Chemo-radiotherapy has a negative impact on the quality of life in NPC patients. This study claims that higher levels of humanistic care were associated with better emotional, cognitive, and social functioning, as well as fewer distressing symptoms, and the reduction of distressing symptoms such as nausea, vomiting, and fatigue. These results show the important impact of humanistic care and provide a basis for future prospective studies on nursing humanistic care for NPC patients, as well as a theoretical basis for exploring how to reduce the physical and mental burden of NPC patients to a certain extent. In addition, nursing humanistic care is also a factor in maintaining a higher overall quality of life during and after treatment, which may inform future supportive care strategies for NPC patients and improve the overall well-being of patients. Future research should improve humanistic care strategies and explore innovative methods to benefit NPC patients in the long term, thus establishing a supportive care system for NPC patients.

## Supplementary Information

Below is the link to the electronic supplementary material.

Supplementary Material 1

## Data Availability

The datasets analysed during the current study are available from the corresponding author on reasonable request.

## Abbreviations

NPC: Nasopharyngeal carcinoma
FACT: The Functional Assessment of Cancer Therapy
EORTC: The European Organisation for Research and Treatment of Cancer
NHCES-NCDP: The Nursing Humanistic Care Experience Scale for Noninfectious Chronic Disease Patients
QLQ–H&N35: The quality of life questionnaire in head and neck cancer
QLQ—C30: The quality of life questionnaire core-30
