# Supplementary material for: Humanistic nursing care and quality of life in patients with nasopharyngeal carcinoma: a prospective observational study
Source: BMC Nurs. 2026 Feb 26;25:304. doi: 10.1186/s12912-026-04455-9 (PMC13040685; doi:10.1186/s12912-026-04455-9)
Supplement: Supplementary file 1 — Supplementary Material 1 [file 12912_2026_4455_MOESM1_ESM.docx]

**Appendix 1.**

The Data Collection Form

**Section A. Demographic Characteristics**

(To be completed at baseline)

1. Participant ID: __________
2. Sex: ☐ Male ☐ Female
3. Age (years): __________
4. Marital status: ☐ Single ☐ Married ☐ Divorced/Widowed
5. Education level: ☐ illiteracy ☐Primary school ☐ Middle school ☐ High and technical secondary school ☐ College/University or above
6. Employment status: ☐ Employed ☐ Unemployed ☐ Retired
7. Place of residence: ☐ Urban ☐ Rural
8. Medical insurance: ☐ Social Basic insurance ☐ Self-pay

**Section B. Clinical Characteristics**

(To be completed from medical records)

1. Primary diagnosis: Nasopharyngeal carcinoma (NPC)
2. TNM stage: ☐ I ☐ II ☐ III ☐ IV
3. Treatment modality: ☐ Induction chemotherapy and concurrent chemotherapy ☐ Concurrent chemoradiotherapy
4. Treatment start date: __________

**Section C. Nursing Humanistic Care Assessment**

(To be completed by the patient)

Instrument used: Nursing Humanistic Care Experience Scale for Noninfectious Chronic Disease Patients (NHCES-NCDP)

- Number of items: 24 items
- Response format: Five-point Likert scale (1 = never, 2 = rarely, 3 = sometimes, 4 = often, 5 = always)
- Subscales:
  - Positive relationship building (Items 1–4, 9–11)
  - Approval and support of choice (Items 12, 14–24)
  - Promoting comfort (Items 5–8, 13)

(Note: The validated Chinese version of the NHCES-NCDP was used. Individual items are not reproduced here due to copyright restrictions.)

**Section D. Quality of Life Assessment – Core Questionnaire**

(To be completed by the patient)

Instrument used: European Organisation for Research and Treatment of Cancer Quality of Life Questionnaire Core-30 (EORTC QLQ-C30)

- Number of items: 30 items
- Domains: 5 functional scales, 3 symptom scales, 1 global health status scale, and 6 single-item symptom measures
- Score range: 0–100

(Higher scores indicate better functioning for functional scales and worse symptoms for symptom scales.)

**Section E. Quality of Life Assessment – Head and Neck Cancer Module**

(To be completed by the patient)

Instrument used: European Organisation for Research and Treatment of Cancer Quality of Life Questionnaire – Head and Neck 35 (EORTC QLQ-H&N35)

- Number of items: 35 items
- Domains: 7 multi-item symptom scales and 11 single-item symptom measures
- Score range: 0–100

(Higher scores indicate more severe symptoms and poorer quality of life.)

**Section F. Data Collection Time Points**

☐ Baseline (before treatment)
☐ Post-treatment (within one week after completion of chemoradiotherapy)

**Section G. Data Collection Method**

- Mode of completion: ☐ Self-administered ☐ Interview-assisted
- Data collector: Trained research nurse
- Data entry: Double-entry and cross-checked for accuracy

**Notes**

All data were collected using standardised questionnaires and a structured case report form. This form was used consistently for all participants to ensure data completeness and uniformity.
